# Supplementary material for: SyNDI: synchronous network data integration framework
Source: BMC Bioinformatics. 2018 Nov 6;19:403. doi: 10.1186/s12859-018-2426-5 (PMC6219086; doi:10.1186/s12859-018-2426-5)
Supplement: Supplementary file 5 — Files of the example presented in Additional file 4. This additional file is a zip package that contains all files used in Additional file 4. Detailed descriptions of each of these files are presented in a README file included in this zip package. (GZ 30411 kb) [file 12859_2018_2426_MOESM5_ESM.gz › SyNDITools/Meme2Fimo/meme_4.9.0_4.tar.gz!/meme_4.9.0/website/html/help_distribution.html]

MEME - Motif Distribution


|  |  |
| --- | --- |
|  | MEME Suite The MEME Suite web application requires the use of JavaScript   Javascript doesn't seem to be available on your browser. MEME searches for similarities among the sequences you give it. How those similarities (motifs) may be shared is determined by the **motif distribution menu**.  1. One per sequence 2. Zero or one per sequence 3. Any number of repetitions  If you choose the first option, MEME assumes that each sequence in the dataset contains **exactly** one occurrence of each motif. This option is the *fastest* and most *sensitive* but the motifs returned by MEME may be "blurry" if any of the sequences is missing them. If you choose the second option, MEME assumes that each sequence may contain **at most** one occurrence of each motif. This option is useful when you suspect that some motifs may be missing from some of the sequences. In that case, the motifs found will be more accurate than using the first option. This option takes more computer time than the first option (about twice as much) and is slightly less sensitive to weak motifs present in all of the sequences.  If you choose the third option, MEME assumes each sequence may contain **any number** of non-overlapping occurrences of each motif. This option is useful when you suspect that motifs repeat multiple times within a single sequence. In that case, the motifs found will be much more accurate than using one of the other options. This option can also be used to discover repeats within a single sequence. This option takes the much more computer time than the first option (about ten times as much) and is somewhat less sensitive to weak motifs which do not repeat within a single sequence than the other two options. |
